# A Comparative Study of the Medial Parapatellar and Midvastus Surgical Approaches for Total Knee Arthroplasty

**DOI:** 10.1055/s-0044-1800945

**Published:** 2025-03-04

**Authors:** Robson Rocha da Silva, Marcos Almeida Matos, Danilo Alves Badaró, Pablo Barreto Prata, Diego Ariel de Lima

**Affiliations:** 1 Departamento de Cirurgia, Escola Bahiana de Medicina e Saúde Pública, Salvador, BA, Brasil.; 2 Santa Casa de Misericórdia da Bahia-Hospital Santa Izabel, Salvador, BA, Brasil.; 3 Departamento de Cirurgia, Hospital Universitário de Lagarto, Lagarto, SE, Brasil.; 4 Departamento de Ciências da Saúde, Universidade Federal Rural do Semi-Árido (UFERSA), Mossoró, RN, Brasil.

**Keywords:** arthroplasty, replacement, knee, knee, quadriceps muscle, range of motion, articular

## Abstract

**Objective** To compare the postoperative recovery outcomes of total knee arthroplasty (TKA) between the medial parapatellar (MP) and midvastus (MV) surgical approaches, focusing on quadriceps strength, knee motion range, and pain.

**Methods** This retrospective study included 82 patients with degenerative knee arthropathy who underwent primary TKA. Patients were divided into two groups: one underwent MP and the other MV. Data were collected on quadriceps strength, knee flexion and extension, pain using the visual analog scale (VAS), and the ability to perform functional tasks like walking and stair climbing. Outcomes were assessed at multiple postoperative intervals.

**Results** Both groups showed improvements in all measured parameters from baseline to the last follow-up, with no significant differences between them in terms of pain and the ability to walk and climb stairs. However, the MV group exhibited statistically significant greater quadriceps strength at the final follow-up.

**Conclusion** While both surgical approaches provided similar overall recovery outcomes, MV led to greater improvements in quadriceps strength and knee extension, suggesting a potentially quicker functional recovery in the early postoperative period.

## Introduction

**Fig. 1:**
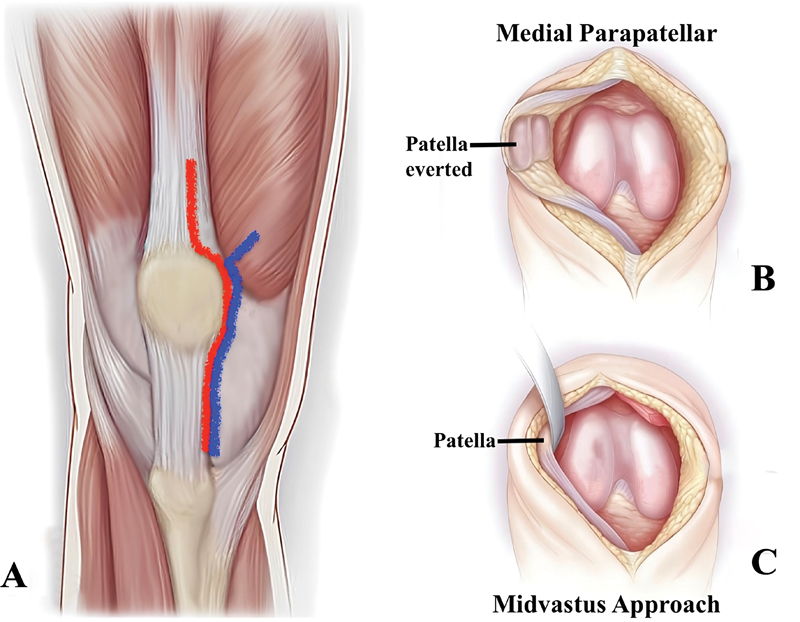
Medial parapatellar and midvastus surgical approaches for total knee arthroplasty. ( **A** ) Medial parapatellar (red line) and midvastus (blue line) surgical approaches for total knee arthroplasty. ( **B** ) Medial parapatellar approach. ( **C** ) Midvastus approach.

Total knee arthroplasty (TKA) is typically performed using the classic medial parapatellar (MP) approach described by Von Langenbeck 1 2 and modified by Insall. 3 This surgical access involves sectioning the quadriceps tendon along the direction of its fibers to gain access to deeper tissues ( Fig. 1 ). Despite the ease and excellent surgical exposure of all structures to be instrumented, a significant blood supply to the patella is interrupted; 2 postoperative recovery is referred to as difficult and often painful, and moreover, the gain in knee flexion is a slow and laborious task in rehabilitation. 4 5

The MP access, despite its great utility, can function as an important factor in the difficulties encountered during the postoperative rehabilitation period. Thus, the gain in muscle strength, recovery of range of motion, and postoperative pain may be consequences of the choice of surgical access used in TKA. 1 6

Other surgical approaches, such as the midvastus (MV), described by Engh, 7 8 have been proposed with the aim of reducing postoperative pain, allowing greater preservation of vascularization, improving patellar stability, quadriceps strength, and facilitating rehabilitation ( Fig. 1 ). This surgical approach is performed by splitting the medial vastus from the superior pole of the patella in the direction of its fibers, allowing a theoretically less aggressive approach to the extensor mechanism of the knee by maintaining the integrity of the quadriceps tendon. 2 9 Although some studies have shown that the MV approach is associated with better outcomes regarding pain, functional recovery, and quadriceps strength, 10 11 others have shown limited benefits and no clinical significance. 12 13

The aim of the present study was to compare the postoperative recovery outcomes of TKA between the MP and MV surgical approaches, focusing on quadriceps strength, knee motion range, and pain.

## Materials and Methods

The study commenced following approval by the Research Ethics Committee. There were no conflicts of interest. This was a retrospective study that evaluated 82 patients who underwent primary TKA due to gonarthrosis. The sample excluded cases of prosthesis revision, cases with extreme preoperative deformities (flexion > 20°, varus > 20°, and valgus > 25°), and those who had undergone previous knee surgeries, such as fracture treatments, osteotomies, or patellectomies. Patients with any neurological or muscular pathology, those with systemic diseases that could interfere with the data results, or obese patients (body mass index > 40) were excluded.

Pre- and postoperative patient data were used, including sociodemographic, clinical, and functional data. The following information was cataloged:

1) Pain grading using the visual analog scale (VAS);
2) The ability for active leg elevation was assessed at 1, 2, 5 (hospital discharge), and 15 days postoperatively, recorded as a binary variable (Yes or No). The elevation capacity of the operated limb was assessed using the Kendall muscle strength grading scale, converted into percentages through the Likert scale; 14
3) Knee flexion and extension range of motion was measured using goniometry, presented in degrees, where full knee extension was considered 0°, any extension deficit was recorded as a negative value, and hyperextension was recorded as a positive value;
4) Walking ability was considered dichotomously, depending on whether the patient was able or unable to walk with support (crutches or walker);
5) The ability to climb a stair step was assessed at 15, 30, 45, and 60 days postoperatively. It was recorded as a binary variable, based on whether the patient could climb at least one 18 cm step using the strength of the operated limb without assistance from the contralateral limb.

All surgeries were performed in the same hospital by the same surgeon and his team. All patients underwent spinal anesthesia (spinal block) and used a tourniquet for bleeding control. In the postoperative period, all patients followed the same analgesia protocol, underwent physiotherapy, and were discharged around the 5 ^th^ postoperative day.

The selected TKA patients were divided into two groups: the first group through the MP approach as described by Von Langenbeck, 1 2 3 and the second through the MV approach as described by Engh. 7 8

Data were presented in the form of descriptive statistics with mean and standard deviation for continuous variables, or absolute and percentage values for categorical variables. For the statistical analysis of this study, the chi-squared or Fisher's exact tests were used when the assessed variable was nominal. For continuous variables, Student's *t* -test was used, and the nonparametric Mann-Whitney test was employed when necessary. We adopted 0.05 as the significance level. Models were also constructed for the serial comparison of variables: knee extension, muscle strength of the operated limb, and the VAS for pain among the groups. For this analysis, repeated measures analysis of variance (ANOVA) was used.

## Results

The sample of this study consisted of 82 patients, divided into the PPM (42 subjects) and MV (40 subjects) groups. The analysis of demographic data was homogeneous between the groups, without any statistically significant difference, except for age, with the MV group patients being slightly older. The other demographic data are found in Table 1 . Over 80% of the data obtained from medical records were considered consistent and reliable and could be used for the purpose of the study.

**Table 1:** Demographic data

	MP	MV	*p* -value
**Gender**			0.265
Female	36 (85.7%)	38 (95%)	
**Age**	65 ± 8.08	69 ± 8.4	
**Diagnosis**			0.025
Osteoarthritis	41 (97.6%)	37 (92.5%)	
Rheumatoid arthritis	1 (2.4%)	2 (5.0%)	
Osteonecrosis	0	1 (2.5%)	0.475
**Side**			
Left	22 (52.4%)	20 (50%)	
Right	20 (47.6%)	20 (50%)	0.829
**Total**	42	40	

**Abbreviations:** MP, medial parapatellar; MV, midvastus.

The ability to elevate the extended limb, to walk, and to climb a step increased progressively in both groups from baseline to the last assessment, with no statistically significant differences in the comparison ( Table 2 ). There was no difference between the groups regarding pain, which showed a trend of improvement ( *p* < 0.100) when comparing the last (15 days) and first assessments ( Table 2 ).

**Table 2:** Postoperative clinical parameters of patients who underwent TKA in the PPM and MV surgical approach groups

Clinical parameter	MP (N = 42)	MV (N = 40)	*p* -value
**Elevation**			
1 day	16 (38.1%)	20 (50%)	0.497
2 days	26 (61.9%)	28 (70%)	0.726
5 days (hospital discharge)	38 (90.5%)	38 (95%)	0.878
15 days	34 (94%)	35 (97%)	0.999
* p* -value*	0.042	0.117	
**Walking**			
2 days	27 (64.3%)	30 (75%)	0.292
5 days (hospital discharge)	42 (100%)	39 (97.5%)	0.488
15 days	35 (97%)	36 (100%)	0.999
* p* -value*	< 0.001	< 0.001	
**Climbing steps**			
15 days	1 (3%)	2 (6%)	0.999
30 days	6 (17%)	9 (25%)	0.562
45 days	14 (39%)	15 (42%)	0.999
60 days	23 (64%)	28 (78%)	0.299
* p* -value*	< 0.001	< 0.001	
**Pain (VAS)**			
1 day	4.05 ± 2.45	4.18 ± 2.65	1.000
5 days (hospital discharge)	1.90 ± 1.44	2.10 ± 1.90	0.522
15 days	2.72 ± 1.86	2.81 ± 1.52	0.837
* p* -value*	0.052	0.082	

**Abbreviations:** MP, medial parapatellar; MV, midvastus; TKA, total knee arthroplasty; VAS, visual analogue scale.

**Note:** * *p* -value within-group, considering the initial moment versus the last assessment.

Muscle strength gradually increased in both groups during the assessment period, however, initially, there was a trend for greater strength in the MV group ( *p* < 0.100) and at the last assessment moment (60 days postoperatively), the MV group had significantly greater strength than the PPM group ( *p* = 0.006), as shown in Table 3 . The ANOVA between the groups for quadriceps muscle strength in elevating the operated limb showed a slight difference in the graphical analysis in favor of the MV approach. In both groups, there was an improvement in the range of motion, with the deficit in extension tending to be smaller in the MV group at the last assessment ( Table 3 ).

**Table 3:** Serial evaluation of postoperative muscle strength and range of motion in the operated limb for the PPM and MV surgical approach groups

Clinical parameter	MP N = 42	MV N = 40	*p* -value
**Muscle strength**			
1 day	29.44 ± 12.17	35.55 ± 15.94	0.072
2 days	35,56 ± 14.43	38.33 ± 13.83	0.407
5 days (hospital discharge)	47.78 ± 11.98	48.89 ± 12.14	0.697
60 days	68.33 ± 12.07	78.67 ± 13.09	0.006
* p* -value*	< 0.001	< 0.001	
**Range of motion**			
**Flexion**			
1 ^st^ day	59.83 ± 22.22°	62.78 ± 22.31°	0.579
5 days (hospital discharge)	84.43 ± 13.27°	87.58 ± 9.86°	0.259
30 days	98.80 ± 14.84°	102.69 ± 10.46°	0.205
* p* -value*	< 0.001	< 0.001	
**Extension**			
1 ^st^ day	−10.57 ± 9.45°	−8.11 ± 7.94°	0.239
5 days (hospital discharge)	−3.71 ± 5.81°	−3.31 ± 4.58°	0.743
30 days	−4.69 ± 6.30°	−2.61 ± 3.61°	0.092
* p* -value*	< 0.001	< 0.001	

**Abbreviations:** MP, medial parapatellar; MV, midvastus.

**Note:** * *p* -value within-group, considering the initial moment versus the last assessment.

We also used combined outcome analysis to assess the potential for patients from each group to present similar results. For this, we selected the following combined effects at the time of the last assessment: mild pain (VAS ≤ 3); being able to climb steps; being able to walk; having knee flexion greater than 90°. Muscle strength was excluded from the combination to avoid bias in favor of one approach, considering that this parameter was significantly better in the MV group. This analysis showed that 11 (26.2%) of the patients in the PPM group presented the combined outcomes versus 20 (50%) in the MV group, with this difference being statistically significant with a *p* -value of 0.026.

The adjusted relative risk (RR) of limb elevation on day-1 postoperatively was 1.31 (confidence interval [CI]: 0.80–2.15), representing a 31% higher probability of elevation on the first postoperative day for patients with MV access. At the time of discharge, this probability (adjusted RR) was 1.05 (CI: 0.93–1.18), representing an effect of 0.5% in favor of the MV access.

## Discussion

This study aimed to compare two patient groups undergoing TKA through the MP and MV surgical approaches, assessing parameters such as pain, early quadriceps strength recovery, extended limb elevation capability, and range of knee motion in the immediate postoperative period. Improvement or a trend toward improvement was noted in all measured parameters across both groups, with no statistically significant differences except for a few instances. Notably, the MV group demonstrated significant muscle strength improvement at the final evaluation. These findings suggest that this approach yields comparable results to the MP approach, with added benefits in quadriceps strength.

Although half of the patients in the MV group were able to elevate the extended limb on the 1 ^st^ postoperative day with a RR of 31%, no significant difference was observed between the two groups at the time of hospital discharge. The ability to walk and climb stairs was similarly comparable between the groups. However, a significant difference was noted in the combined outcomes analysis, favoring the MV group, which showed better levels of pain, knee flexion, walking ability, and stair climbing capability. This suggests that improved combined variable levels might translate into noticeable benefits for basic daily activities involving the lower limb in the early postoperative phase.

Over the past 30 years, various surgical approaches have been proposed for TKA, including the MP, MV, and subvastus approaches. 1 The MV approach, as described by Engh et al., 7 was introduced as an alternative to the MP approach, with several theoretical advantages such as reduced damage to the extensor mechanism by preserving the quadriceps tendon. 15

In assessing muscle strength in the operated limb, the ability to elevate the leg with an extended knee was used as a marker of early functional quadriceps recovery. This test's capability immediately postoperative has also been utilized by other authors as an indicator of the return of quadricep function. 4 White et al. 16 reported that patients undergoing the MV approach had a better ability to elevate the extended leg after eight days postsurgery. A similar observation was made in a prospective study that analyzed outcomes from 24 bilateral TKAs, noting that patients who underwent the MV approach could elevate the extended leg in 1.7 days postoperatively as opposed to 5.2 days in the PPM group. 10

Bäthis et al., 11 using an isokinetic dynamometer for late quadriceps strength evaluation, observed better and quicker recovery with the MV approach. Another prospective, randomized, double-blind study utilizing isokinetic evaluation found that quadriceps extension strength was higher in the MV group than in the PPM group three weeks post-surgery; this difference became statistically insignificant after 6 weeks. 12

The combination of limb elevation capability, quadriceps muscle strength, and improved motion range may collectively facilitate the lower limb function recovery after surgery. Nutton et al. 17 observed earlier recovery of extended limb elevation and crutch-assisted walking in patients undergoing the MV approach, with walking ability enhanced by dynamic balance and quadriceps strength. Maestro et al., 9 despite noting more preoperative flexion deformities in knees undergoing the MV approach, found better range of motion and greater active extension in these patients postsurgery. The overall improvement in range of motion during the initial weeks postsurgery was confirmed in meta-analyses by Liu et al. 18 and Alcelik et al., 19 highlighting the primary advantages of the MV over the PPM approach for TKA. Similarly, Aslam et al. 20 needed fewer retinacular releases, could perform the limb elevation test earlier, and observed less extension deficit in the first postoperative week. These literature findings corroborate the results from the combined analysis of this study, reinforcing the hypothesis of potentially earlier functional recovery in the group operated through the MV approach.

## Limitations

This study's retrospective nature implies some reliability loss in data obtained from medical records that, in our case, were not digitized. Therefore, any record with erasures, missing pages, or even missing sheets was excluded. As a result, we obtained 80% reliable records. The absence of randomization might have introduced some bias, despite the apparent homogeneity between the groups. Another limitation of the present study was that the comparison of prosthetic component positioning was not performed, as the MV approach generally does not allow the same exposure and ease for positioning guides and making bone cuts. Objective assessments, such as dynamometry or gait analysis, might offer more accurate estimations than subjective clinical evaluations (walking ability, stair-climbing capability, or VAS-graded strength). However, the assessment type used in this study is more applicable in real clinical settings than more complex tests, which might not be routinely feasible in most clinics and hospitals.

## Conclusion

While both surgical approaches provided similar overall recovery outcomes, MV led to greater improvements in quadriceps strength and knee extension, suggesting a potentially quicker functional recovery in the early postoperative period.
