# Supplementary material for: A Comparative Study of the Medial Parapatellar and Midvastus Surgical Approaches for Total Knee Arthroplasty
Source: Rev Bras Ortop (Sao Paulo). 2025 Mar 4;60(1):s00441800945. doi: 10.1055/s-0044-1800945 (PMC11879484; doi:10.1055/s-0044-1800945)
Supplement: Related texts

# Research article: Estudo comparativo das abordagens cirúrgicas parapatelar medial e midvasto para artroplastia total do joelho

**Keywords:** amplitude de movimento articular, artroplastia do joelho, joelho, músculo quadríceps

## Abstract

**Objetivo** Comparar os resultados da recuperação pós-operatória da artroplastia total do joelho (ATJ) entre as abordagens cirúrgicas parapatelar medial (PPM) e midvasto (MV), enfocando a força do quadríceps, a amplitude de movimento do joelho e a dor.

**Métodos** Este estudo retrospectivo incluiu 82 pacientes com artropatia degenerativa do joelho que foram submetidos à ATJ primária. Os pacientes foram divididos em dois grupos: um submetido à PPM e o outro à MV. Foram coletados dados sobre a força do quadríceps, flexão e extensão do joelho, dor segundo a escala visual análoga (EVA) e a capacidade de realizar tarefas funcionais, como caminhar e subir escadas. Os resultados foram avaliados em vários intervalos pós-operatórios.

**Resultados** Ambos os grupos mostraram melhorias em todos os parâmetros medidos desde a linha de base até o último acompanhamento, sem diferenças significativas entre eles em termos de dor e a capacidade de caminhar e subir escadas. No entanto, o grupo MV exibiu uma força do quadríceps significativamente maior na última avaliação.

**Conclusão** Embora ambas as abordagens cirúrgicas tenham fornecido resultados de recuperação geral semelhantes, a MV levou a maiores melhorias na força do quadríceps e extensão do joelho, sugerindo uma recuperação funcional mais rápida no período pós-operatório inicial.

## Introdução

A artroplastia total do joelho (ATJ) é normalmente realizada usando a abordagem clássica parapatelar medial (PPM) descrita por Von Langenbeck 1 2 e modificada por Insall. 3 Esta cirurgia envolve a secção do tendão do quadríceps ao longo da direção de suas fibras para obter acesso aos tecidos mais profundos ( Fig. 1 ). Apesar da facilidade e excelente exposição cirúrgica de todas as estruturas a serem instrumentadas, há interrupção significativa do suprimento sanguíneo para a patela; 2 a recuperação pós-operatória é relatada como difícil e muitas vezes dolorosa e, além disso, o ganho de flexão do joelho é lento e trabalhoso durante a reabilitação. 4 5

**Fig. 1:** Abordagens cirúrgicas para artroplastia total do joelho. ( **A** ) Abordagens cirúrgicas parapatelar medial (linha vermelha) e midvasto (linha azul) para artroplastia total do joelho. ( **B** ) Abordagem parapatelar medial. ( **C** ) Abordagem midvasto.

A abordagem PPM, apesar de sua grande utilidade, pode ser um fator importante nas dificuldades encontradas durante o período de reabilitação pós-operatória. Assim, o ganho de força muscular, a recuperação da amplitude de movimento e a dor pós-operatória podem ser consequências da escolha da abordagem cirúrgica utilizada na ATJ. 1 6

Outras abordagens cirúrgicas, como o midvasto (MV), descrito por Engh, 7 8 têm sido propostas com o objetivo de reduzir a dor pós-operatória, permitir a maior preservação da vascularização, melhorar a estabilidade patelar e a força do quadríceps e facilitar a reabilitação ( Fig. 1 ). Esta abordagem cirúrgica é realizada pela divisão do vasto medial do polo superior da patela na direção de suas fibras, permitindo uma abordagem teoricamente menos agressiva ao mecanismo extensor do joelho e mantendo a integridade do tendão do quadríceps. 2 9 Embora alguns estudos tenham demonstrado que a abordagem MV está associada a melhores resultados em relação à dor, recuperação funcional e força do quadríceps, 10 11 outros mostraram benefícios limitados e sem importância clínica significativa. 12 13

O objetivo do presente estudo foi comparar os resultados da recuperação pós-operatória da ATJ entre as abordagens cirúrgicas PPM e MV com foco na força do quadríceps, na amplitude de movimento do joelho e na dor.

## Materiais e Métodos

O estudo teve início após aprovação do Comitê de Ética em Pesquisa. Não houve conflitos de interesse. Este foi um estudo retrospectivo com avaliação de 82 pacientes submetidos à ATJ primária devido à gonartrose. A amostra excluiu casos de revisão de prótese, com deformidades pré-operatórias extremas (flexão > 20°, varo > 20° e valgo > 25°) e que haviam passado por cirurgias prévias no joelho, como tratamentos de fraturas, osteotomias ou patelectomias. Pacientes com qualquer doença neurológica ou muscular, doenças sistêmicas que pudessem interferir nos resultados dos dados ou obesos (índice de massa corporal > 40) foram excluídos.

Foram utilizados dados pré e pós-operatórios dos pacientes, incluindo dados sociodemográficos, clínicos e funcionais. As seguintes informações foram registradas:

1) Classificação da dor usando a escala visual analógica (EVA);
2) Avaliação da capacidade de elevação ativa da perna aos 1, 2, 5 (alta hospitalar) e 15 dias pós-operatório, registrada como variável binária (Sim ou Não). A capacidade de elevação do membro operado foi avaliada usando a escala de classificação de força muscular de Kendall, convertida em porcentagens por meio da escala de Likert; 14
3) Medida da amplitude de movimento de flexão e extensão do joelho por goniometria, apresentada em graus, em que a extensão total do joelho foi considerada 0°, qualquer déficit de extensão foi registrado como um valor negativo e a hiperextensão foi registrada como um valor positivo;
4) Consideração dicotômica da capacidade de caminhar, dependendo se o paciente conseguia ou não caminhar com apoio (muletas ou andador);
5) Avaliação da capacidade de subir um degrau de escada aos 15, 30, 45 e 60 dias pós-operatórios. Esta capacidade foi registrada como uma variável binária, com base em se o paciente conseguia subir pelo menos um degrau de 18 cm usando a força do membro operado sem assistência do membro contralateral.

Todas as cirurgias foram realizadas no mesmo hospital e pelo mesmo cirurgião e sua equipe. Todos os pacientes foram submetidos à raquianestesia (bloqueio espinhal) e usaram torniquete para controle de sangramento. No período pós-operatório, todos os pacientes seguiram o mesmo protocolo de analgesia, foram submetidos à fisioterapia e receberam alta por volta do 5° dia após o procedimento.

Os pacientes submetidos à ATJ selecionados foram divididos em dois grupos: o primeiro grupo pela abordagem PPM descrita por Von Langenbeck 1 2 3 e o segundo pela abordagem MV descrita por Engh. 7 8

Os dados foram apresentados na forma de estatística descritiva como média e desvio-padrão para variáveis contínuas ou valores absolutos e percentuais para variáveis categóricas. A análise estatística deste estudo utilizou os testes de qui-quadrado ou exato de Fisher quando a variável avaliada era nominal. Para variáveis contínuas, foi usado o teste *t* de Student e o teste não paramétrico de Mann-Whitney foi empregado quando necessário. Foi adotado 0,05 como nível de significância. Também foram construídos modelos para comparação seriada das variáveis: extensão do joelho, força muscular do membro operado e EVA de dor entre os grupos. Para esta análise, foi utilizada a análise de variância (ANOVA) para medidas repetidas.

## Resultados

A amostra deste estudo foi composta por 82 pacientes, divididos nos grupos PPM (42 indivíduos) e MV (40 indivíduos). A análise dos dados demográficos foi homogênea entre os grupos, sem diferença estatisticamente significativa, exceto quanto à idade, já que os pacientes do grupo MV eram um pouco mais velhos. A Tabela 1 mostra os demais dados demográficos. Mais de 80% dos dados obtidos dos prontuários médicos foram considerados consistentes e confiáveis e puderam ser usados para o propósito do estudo.

**Tabela 1:** Dados demográficos

	PPM	MV	Valor de *p*
**Sexo**			0,265
Feminino	36 (85,7%)	38 (95%)	
**Idade**	65 ± 8,08	69 ± 8,4	
**Diagnóstico**			0,025
Osteoartrite	41 (97,6%)	37 (92,5%)	
Artrite reumatoide	1 (2,4%)	2 (5,0%)	
Osteonecrose	0	1 (2,5%)	0,475
**Lado**			
Esquerdo	22 (52,4%)	20 (50%)	
Direito	20 (47,6%)	20 (50%)	0,829
**Total**	42	40	

**Abreviações:** PPM, parapatelar medial; MV, midvasto.

A capacidade de elevar o membro estendido, de caminhar e de subir um degrau aumentou progressivamente em ambos os grupos desde o início até a última avaliação, sem diferenças estatisticamente significativas à comparação ( Tabela 2 ). Não houve diferença entre os grupos quanto à dor, que apresentou tendência de melhora ( *p* < 0,100) quando a última avaliação (15 dias) foi comparada à primeira ( Tabela 2 ).

**Tabela 2:** Parâmetros clínicos pós-operatórios de pacientes submetidos à artroplastia total do joelho nos grupos de abordagem cirúrgica PPM e MV

Parâmetro clínico	PPM N = 42	MV N = 40	Valor de *p*
**Elevação**			
1 dia	16 (38,1%)	20 (50%)	0,497
2 dias	26 (61,9%)	28 (70%)	0,726
5 dias (alta hospitalar)	38 (90,5%)	38 (95%)	0,878
15 dias	34 (94%)	35 (97%)	0,999
Valor de *p* *	0,042	0,117	
**Caminhar**			
2 dias	27 (64,3%)	30 (75%)	0,292
5 dias (alta hospitalar)	42 (100%)	39 (97,5%)	0,488
15 dias	35 (97%)	36 (100%)	0,999
Valor de *p* *	< 0,001	< 0,001	
**Subir escadas**			
15 dias	1 (3%)	2 (6%)	0,999
30 dias	6 (17%)	9 (25%)	0,562
45 dias	14 (39%)	15 (42%)	0,999
60 dias	23 (64%)	28 (78%)	0,299
Valor de *p* *	< 0,001	< 0,001	
**Dor (EVA)**			
1 dia	4,05 ± 2,45	4,18 ± 2,65	1,000
5 dias (alta hospitalar)	1,90 ± 1,44	2,10 ± 1,90	0,522
15 dias	2,72 ± 1,86	2,81 ± 1,52	0,837
Valor de *p* *	0,052	0,082	

**Abreviações:** EVA, escala visual análoga; PPM, parapatelar medial; MV, midvasto.

**Nota:** *Valor de *p* entre grupos considerando a primeira e a última avaliação.

A força muscular aumentou gradativamente em ambos os grupos durante o período de avaliação. A princípio, porém, houve uma tendência de maior força no grupo MV ( *p* < 0,100) e, no último momento de avaliação (60 dias após a cirurgia), o grupo MV apresentou força significativamente maior que o grupo PPM ( *p* = 0,006), como visto na Tabela 3 . A ANOVA entre os grupos para força do músculo quadríceps na elevação do membro operado mostrou uma pequena diferença na análise gráfica em favor da abordagem MV. Em ambos os grupos, houve melhora na amplitude de movimento, com o déficit de extensão tendendo a ser menor no grupo MV à última avaliação ( Tabela 3 ).

**Tabela 3:** Avaliação seriada da força muscular e amplitude de movimento pós-operativos do membro operado nos grupos submetidos à abordagem cirúrgica PPM e MV

Parâmetro clínico	PPM N = 42	MV N = 40	Valor de *p*
**Força muscular**			
1 dia	29,44 ± 12,17	35,55 ± 15,94	0,072
2 dias	35,56 ± 14,43	38,33 ± 13,83	0,407
5 dias (alta hospitalar)	47,78 ± 11,98	48,89 ± 12,14	0,697
60 dias	68,33 ± 12,07	78,67 ± 13,09	0,006
Valor de *p* *	< 0,001	< 0,001	
**Amplitude de movimento**			
**Flexão**			
1 dia	59,83 ± 22,22°	62,78 ± 22,31°	0,579
5 dias (alta hospitalar)	84,43 ± 13,27°	87,58 ± 9,86°	0,259
30 dias	98,80 ± 14,84°	102,69 ± 10,46°	0,205
Valor de *p* *	< 0,001	< 0,001	
**Extensão**			
1 dia	−10,57 ± 9,45°	−8,11 ± 7,94°	0,239
5 dias (alta hospitalar)	−3,71 ± 5,81°	−3,31 ± 4,58°	0,743
30 dias	−4,69 ± 6,30°	−2,61 ± 3,61°	0,092
Valor de *p* *	< 0,001	< 0,001	

**Abreviações:** PPM, parapatelar medial; MV, midvasto.

**Nota:** *Valor de *p* considerando a primeira e a última avaliação.

Também utilizamos a análise de desfechos combinados para avaliação da possibilidade de os pacientes de cada grupo apresentarem resultados semelhantes. Para isso, selecionamos os seguintes efeitos combinados no momento da última avaliação: dor branda (EVA ≤ 3); capacidade de subir degraus; capacidade de caminhar; e apresentar flexão do joelho acima de 90°. A força muscular foi excluída da combinação para evitar viés em favor de uma abordagem, considerando que esse parâmetro foi significativamente melhor no grupo MV. Essa análise mostrou que 11 (26,2%) dos pacientes do grupo PPM apresentaram desfechos combinados em comparação a 20 (50%) no grupo MV; esta diferença foi estatisticamente significativa, com um valor de *p* de 0,026.

O risco relativo (RR) ajustado de elevação do membro no 1° dia pós-operatório foi de 1,31 (intervalo de confiança [IC]: 0,80–2,15), representando uma probabilidade 31% maior de elevação no 1° dia pós-operatório nos pacientes submetidos à abordagem MV. À alta, essa probabilidade (RR ajustado) foi de 1,05 (IC: 0,93–1,18), representando um efeito de 0,5% a favor da abordagem MV.

## Discussão

Este estudo teve como objetivo comparar dois grupos de pacientes submetidos à ATJ por meio das abordagens cirúrgicas PPM e MV, avaliando parâmetros como dor, recuperação precoce da força do quadríceps, capacidade de elevação estendida do membro e amplitude de movimento do joelho no período pós-operatório imediato. Melhora ou tendência à melhora foi observada em todos os parâmetros medidos em ambos os grupos, sem diferenças estatisticamente significativas, exceto em alguns casos. Notavelmente, o grupo MV demonstrou melhora significativa da força muscular à avaliação final. Esses achados sugerem que essa abordagem produz resultados comparáveis à PPM, com mais benefícios na força do quadríceps.

Embora metade dos pacientes do grupo MV tenham conseguido elevar o membro estendido no 1° dia pós-operatório com RR de 31%, nenhuma diferença significativa foi observada entre os dois grupos à alta hospitalar. A capacidade de andar e subir escadas foi similarmente comparável entre os grupos. No entanto, uma diferença significativa foi notada na análise de desfechos combinados, favorecendo o grupo MV, que apresentou melhores níveis de dor, flexão do joelho e capacidade de andar e subir escadas. Isso sugere que melhores níveis variáveis combinados podem se traduzir em benefícios perceptíveis em atividades diárias básicas envolvendo o membro inferior na fase pós-operatória inicial.

Nos últimos 30 anos, várias abordagens cirúrgicas foram propostas para ATJ, incluindo as abordagens PPM, MV e subvasto. 1 A abordagem MV descrita por Engh et al. 7 foi introduzida como uma alternativa à PPM com várias vantagens teóricas, como redução de danos ao mecanismo extensor devido à preservação do tendão do quadríceps. 15

Na avaliação da força muscular no membro operado, a capacidade de elevar a perna com o joelho estendido foi usada como um marcador da recuperação funcional precoce do quadríceps. A capacidade desse teste imediatamente pós-operatório também foi utilizada por outros autores como um indicador do retorno da função do quadríceps. 4 White et al. 16 relataram que pacientes submetidos à abordagem MV tiveram uma melhor capacidade de elevar a perna estendida 8 dias após a cirurgia. Uma observação semelhante foi feita em um estudo prospectivo que analisou os resultados de 24 ATJs bilaterais, em que os pacientes submetidos à abordagem MV puderam elevar a perna estendida em 1,7 dias após o procedimento em comparação a 5,2 dias no grupo PPM. 10

Bäthis et al., 11 usando um dinamômetro isocinético para avaliação tardia da força do quadríceps, observaram uma recuperação melhor e mais rápida com a abordagem MV. Outro estudo prospectivo, randomizado e duplo-cego utilizando avaliação isocinética descobriu que a força de extensão do quadríceps foi maior no grupo MV do que no grupo PPM 3 semanas após a cirurgia; essa diferença se tornou estatisticamente insignificante depois de 6 semanas. 12

A combinação da capacidade de elevação dos membros, da força do músculo quadríceps e da maior amplitude de movimento pode, coletivamente, facilitar a recuperação da função dos membros inferiores após a cirurgia. Nutton et al. 17 observaram recuperação mais rápida da elevação estendida do membro e da caminhada assistida por muletas em pacientes submetidos à abordagem MV, com maior capacidade de andar devido ao equilíbrio dinâmico e força do quadríceps. Maestro et al., 9 apesar de notar mais deformidades de flexão pré-operatórias em joelhos submetidos à abordagem MV, notaram maior amplitude de movimento e maior extensão ativa nesses pacientes após a cirurgia. A melhora geral na amplitude de movimento durante as primeiras semanas após a cirurgia foi confirmada em meta-análises por Liu et al. 18 e Alcelik et al., 19 destacando as principais vantagens da abordagem MV sobre a abordagem PPM na ATJ. Da mesma forma, Aslam et al. 20 necessitaram de menos liberações retinaculares, puderam realizar o teste de elevação do membro mais cedo e observaram menor déficit de extensão na primeira semana pós-operatória. Esses achados da literatura corroboram os resultados da análise combinada deste estudo, reforçando a hipótese de recuperação funcional possivelmente mais precoce no grupo submetido à abordagem MV.

## Limitações

A natureza retrospectiva deste estudo implica alguma perda de confiabilidade nos dados obtidos de prontuários médicos que, em nosso caso, não são digitalizados. Portanto, qualquer registro com rasuras ou páginas faltantes foi excluído. Como resultado, obtivemos 80% de registros confiáveis. A ausência de randomização pode ter introduzido algum viés apesar da aparente homogeneidade entre os grupos. Outra limitação do presente estudo foi que a ausência de comparação do posicionamento do componente protético, pois a abordagem MV geralmente não permite a mesma exposição e facilidade para posicionamento de guias e secções ósseas. Avaliações objetivas, como dinamometria ou análise da marcha, podem oferecer estimativas mais precisas do que avaliações clínicas subjetivas (capacidade de caminhar, capacidade de subir escadas ou força graduada pela EVA). No entanto, o tipo de avaliação usado neste estudo é mais aplicável em ambientes clínicos reais do que testes mais complexos, que podem não ser viáveis na rotina da maioria das clínicas e dos hospitais.

## Conclusão

Embora as duas abordagens cirúrgicas tenham proporcionado resultados gerais de recuperação semelhantes, a MV levou a maiores melhoras na força do quadríceps e na extensão do joelho, sugerindo uma recuperação funcional possivelmente mais rápida no período pós-operatório inicial.
